# Link-prediction to tackle the boundary specification problem in social network surveys

**DOI:** 10.1371/journal.pone.0176094

**Published:** 2017-04-20

**Authors:** Tobias Jordan, Oto Costa Pinho Alves, Philippe De Wilde, Fernando Buarque de Lima-Neto

**Affiliations:** 1 School of Computing, University of Kent, Canterbury, Kent, United Kingdom; 2 Computational Intelligence Research Group, Universidade de Pernambuco, Recife, Pernambuco, Brazil; Universidad Nacional de Mar del Plata, ARGENTINA

## Abstract

Diffusion processes in social networks often cause the emergence of global phenomena from individual behavior within a society. The study of those global phenomena and the simulation of those diffusion processes frequently require a good model of the global network. However, survey data and data from online sources are often restricted to single social groups or features, such as age groups, single schools, companies, or interest groups. Hence, a modeling approach is required that extrapolates the locally restricted data to a global network model. We tackle this Missing Data Problem using Link-Prediction techniques from social network research, network generation techniques from the area of Social Simulation, as well as a combination of both. We found that techniques employing less information may be more adequate to solve this problem, especially when data granularity is an issue. We validated the network models created with our techniques on a number of real-world networks, investigating degree distributions as well as the likelihood of links given the geographical distance between two nodes.

## Introduction

Social networks shape our lives [1]. They play a major role in the diffusion of ideas, norms, information, behaviors or viruses [1–4]. This motivates a wide range of research concerning social networks, frequently triggered by the emergence of online social networks and large data sets containing relational data. However, when it comes to close personal relations such as affective contacts or close friendships and especially when detailed behavioral data is to be collected, surveys of individuals are still required. Those studies and, to a lesser extent also data gathered from online sources, generally suffer from missing data. Missing data may stem from a survey design, restricted to a certain type of participants, to certain relations between participants or from the focus on certain places such as schools, classes, companies or offices. This issue is also referred to as the “boundary specification problem” [5]. Another persisting problem in social network surveys is the restriction of the number of contacts to choose. This “fixed choice effect” [6] appears when survey participants are asked to nominate a certain number of contacts. Surveys including a large number of participants may not be capable of capturing relations that could exist between survey participants from distinct places or of different types, such as pupils from different schools or employees from different companies. Hence those large social network surveys often appear to consist of many disconnected components. The availability of a large volume of network data creates the possibility to investigate local network effects based on a large number of empirical data. However, in large societies some trends, behaviors or norms may emerge in one part of the society and then diffuse to other parts. There may be local circumstances in one component that prevent individuals within that component from adopting whatever is spreading throughout the network and hence impede it from becoming a global trend. Moreover there is evidence that network structure and network heterogeneity heavily affect global behavioral outcomes of network diffusion processes [7, 8]. Therefore, in order to understand and simulate global network effects on a population or society level, it is desirable to develop mechanisms that indicate reasonable connections between those isolated components or between network components from distinct surveys. Hereby it may be possible to create a model of the global network that features real-world properties. In this paper we investigate the performance of techniques for network generation [9] and link-prediction [10], as well as a combination of both, in filling up the informational gap that frequently occurs between isolated components in social network surveys. We are explicitly not aiming at creating very precise estimations of links, for our purpose a conclusive model of the global network is required.

### Background

The following literature review reveals how the problem of missing information in social network data has been tackled from different disciplines. Missing information may be classified as missing completely at random (MCAR) if the missing value does neither depend on other missing values, nor on observable values, missing at random (MAR) if the missing value does not depend on other missing values and missing not at random (MNAR) when the reason for the missing information can be found in the information itself [11]. We find that there are several fairly well performing methods to deal with both, (i) the total absence of information about links between individuals in the network (MNAR), and (ii) the randomly missing information about links within a network (MAR) or (MCAR). Nevertheless, to the best of our knowledge it has not been studied yet how systematically missing data between isolated components from social network surveys (MNAR) may be inferred (or imputed) in order to enable simulations on a global network model. We denote this type of missing data as missing not at random (MNAR), since the missing information about an existing link between two nodes may depend on the nominated friend node: if the friend is included in the boundary specifications, the link is being recorded, if not the information gets lost.

#### Missing data in the social sciences

Traditional solutions to the missing data problems “Fixed Choice Effect” and “Boundary Specification Problem” are applied in survey planning, dealing with the careful definition of the survey group [5, 6, 12].

The “Fixed Choice Effect” seems to disturb associativity measures and degree distributions which may explain the frequent deviation of those measures when comparing surveyed social networks to other known social networks [12]. The problem of missing data after completing data collection has been approached by social sciences mainly under the term imputation [13]. The set of applied mechanisms incorporates for example the estimation of missing reciprocal ties in directed networks (reconstruction) [14], the replacement of incomplete respondents by similar alters (hot deck imputation) [13], or using the concept of preferential attachment (assortativity) [15].

#### Link-prediction in complex networks

The missing data problem for social networks is a recent issue for researchers dealing with large social networks from online sources. Here links may be omitted due to privacy restrictions, or missing because observed networks tend to be dynamic. The task to predict links to be established in the future or links that have been omitted due to other reasons is here called the “Link Prediction Problem” [10]. Unsupervised measures for link prediction build on the “similarity” of nodes in terms of network properties as for example the number of common neighbors or draw from common properties of social networks such as assortativity [10]. Other approaches are for example supervised random walks [16], methods based on community structure [17] or on mutual information [18]. Furthermore, the problem to predict links between individuals that are not part of the same data set or platform has been successfully tackled using machine learning techniques such as classifier systems [19].

A special case of the “Link-Prediction Problem” occurs when no previous data of the network is available, and the network structure is to be re-build based on other information about the nodes. This problem may arise in co-purchasing networks or recommendation networks where information about the nodes is available, but connections between them are omitted or simply not informed [20]. This special situation requires a different approach for link-prediction, since network based measures are not applicable due to the total absence of links. A well performing mechanism to tackle this problem is a two phase bootstrapping method [20]. Here a bootstrap probabilistic graph is being estimated from the node properties in a first step, assigning a probability for the existence of each possible link in the network. Subsequently network based measures are applied to the bootstrap probabilistic graph in order to reinforce the probability of links to exist. Finally, the researcher defines a probability threshold *t* so that all links with probability *p* ≥ *t* are estimated as existing links. For a review of link-prediction techniques see [21].

#### Network generation

Scientists in the fields of Complex Network Sciences and Social Simulation have extensively studied models to create networks featuring characteristics that can be found in real complex networks. Especially in social networks there is consensus that models that generate social network representations should assure that generated networks feature limited size and heterogeneous right-skewed distribution of degree allowing for a “cut-off’ for higher degrees in case of close relationships [22]. The networks should furthermore incorporate high clustering, low density, positive assortativity by degree, and short path lengths [23]. Literature about social networks of students suggests that pupils should have lots of contacts in their own classroom and fewer within other classrooms. Furthermore, this pattern may be found at larger scales i.e, many contacts within school, fewer contacts between schools [24]. Moreover, recent studies with location-based social networks suggest that on a global scale, distance matters for the likelihood of the existence of links between any two nodes [25]. Those studies further suggest that the relation between link-probability *P*(*l*) and distance of any two nodes follows approximately a law *P*(*l*) *d*^−*α*^, where the exponent *α* lies between 0.5 and 2 for different networks [26, 27].

An approach to generate complex networks has been the use of random graph theory [28]. However, those graphs failed to exhibit the scale-free property or to have right-skewed degree distributions [29]. Models to generate networks with more plausible features from scratch have been developed on the ground of preferential attachment, where the probability of a new vertex being attached to an existing vertex depends on the degree of the existing vertex [29–31]. Other approaches focus on local interaction of nodes and equip the models with mechanisms that represent human behavior such as inviting and visiting each other [32] or incorporate the idea of social proximity and agents moving and meeting within a “social space” [33]. The idea of social proximity is also an essential part of the social circle model for generating large artificial social networks for social simulations [23]. Here a “Social Reach” is defined, allowing the agents to connect to other agents within their “Social Reach”’, when the relation reciprocates. The “Social Reach” may be interpreted as a social distance, set for example by the number of common friends or the similarity of interests between two agents, but can also be interpreted as a physical distance like for example the distance between the domiciles of two individuals. It is established that this approach enables the researcher to create networks from scratch that exhibit characteristics that match the aforementioned network properties. Similar to this is the “Waxman model” [34] which does not define a fixed social reach, but employs an exponential decay model to create links depending on the local proximity of nodes.

## Analysis: Problem description and link prediction approaches

### Problem

**Fig 1:**
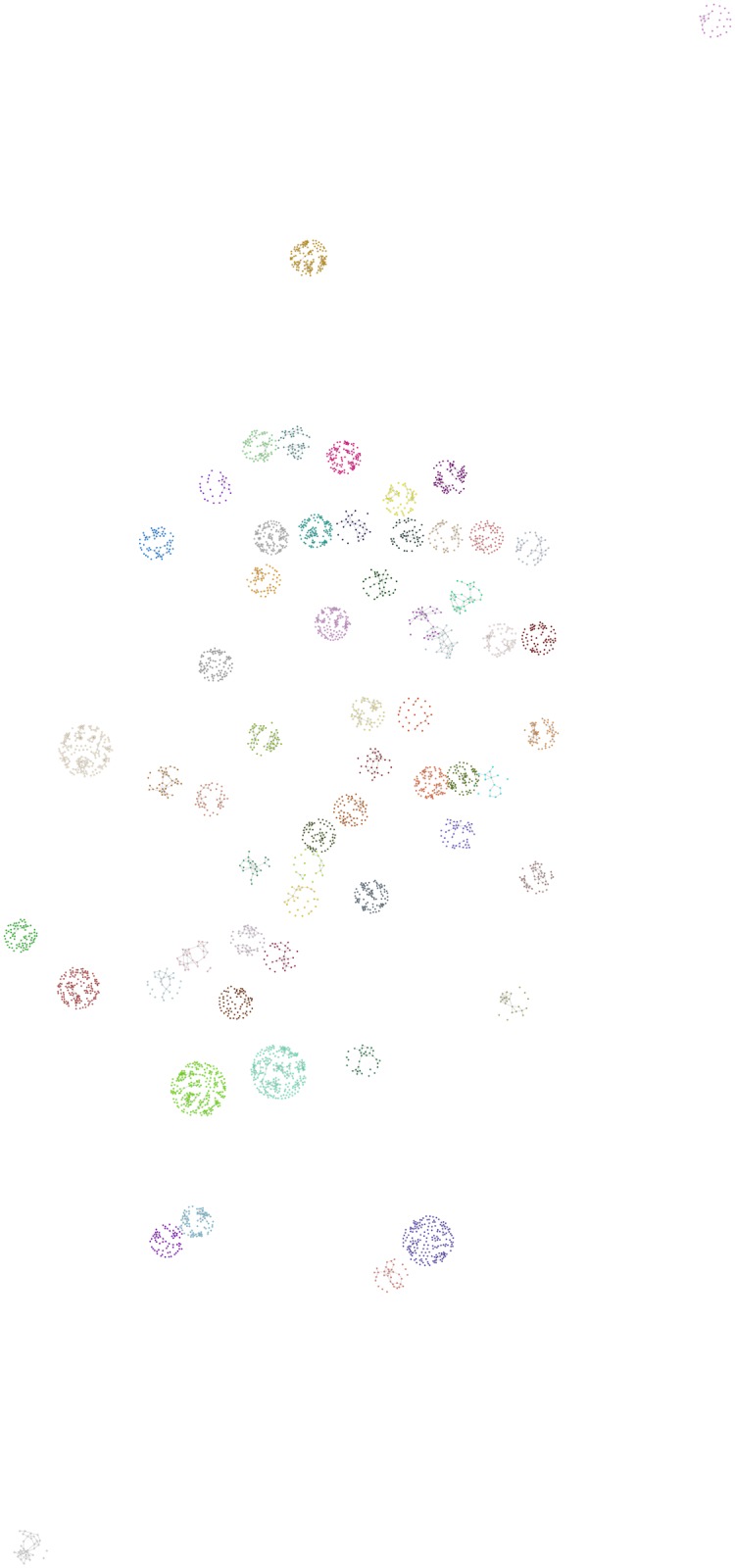
School-clusters from Recife. The Figure presents the individual pupils that participated in the FUNDAJ-Survey. Pupils are colored according to their school. Location of pupils is assigned by a Fruchtermann-Reingold algorithm [36] centered around the location of their school within the city of Recife. Grey lines indicate friendships between pupils as registered by the survey. As social networks where solely surveyed within schools, isolated components appear for each school.

The social network we are analyzing is located in the Brazilian city of Recife. The problem context is described in “*Determinantes do desempenho escolar na rede de ensino fundamental do Recife*” [35]. The study has the objective to estimate a linear hierarchic model in order to quantify the effect that schooling infrastructure projects have on the performance of school children. The survey was conducted by Fundação Joaquim Nabuco (FUNDAJ) in 2013, gathering data from more than 4000 pupils in public schools in the northeastern Brazilian city Recife. The data contain among others the social network of the pupils and their performance in the subject of maths at the beginning and at the end of the year. Children were asked to nominate their five best friends. If the friends went to the same class, a questionnaire was sent to these students as well. In this way, a network containing 4191 students was generated. However, since the friendship nominations were only traced if the students were within the same class, the network is subdivided in 219 disconnected components from 122 schools. The schools are distributed over the districts of Recife according to population size of the respective district. Hence, very large districts are represented by more schools than smaller districts. Fig 1 illustrates the distribution of the isolated components throughout the city of Recife. The pupils of each school are represented by points, where pupils from the same school are assigned equal color. The location of the pupils is defined by a Fruchtermann-Reingold algorithm [36], centered around the location of their school on the map. The area of the graph is hereby given as *n*^2^, where *n* denotes the number of pupils of the respective school.

Recife, as most large Brazilian cities, is characterized by a marked social divide between districts [37]. Hence districts and the people living in them are differently affected by governmental social welfare programs as for example the “Bolsa Familia” Program [38]. In order to simulate indirect effects that stem from those social welfare programs, for example the spreading of behavior through friendship or kinship networks, a model of the global structure of the social networks of the population is required. The following Sections present and compare different approaches to deal with this missing data problem that appears frequently due to boundary specification in social network surveys.

### Approaches

We employ three different approaches to impute friendship connections between the isolated components of the original semi-connected network. Hereby it is important to note that we aim at creating a good model of the global network rather than actually finding the individual connections. The first approach stems from the “Social Circles” model proposed by Hamill and Gilbert [23], second we employ a Link-Prediction technique based on a two phase bootstrapping procedure. We finally join elements from both techniques in a combined approach.

#### Social circles/Waxman model approach

Our approach to the problem described in the previous paragraph, was inspired by Hamill and Gilbert’s social circle model [23]. Hereby we define the social distance as the physical distance between the schools of two pupils. However, unlike Hamill and Gilbert, our challenge is not to create a social network from scratch, but to insert links between the isolated components of our already existing social network data set. Hence we had to adapt the social circles approach to our underlying problem. As we possess the location of the individuals only on district level, a stand-alone social circles approach would lead to very densely connected components because all individuals that went to school in the same district were within one social circle and hence connected to each other. Thus, instead of purely adopting the social radius, we decided to use an exponential decay model based on a probability function related to the social distance, which approximates our approach to the well known “Waxman Model” [34]. In contrast do Hamill and Gilbert’s work we introduce heterogeneity of the span of social networks not by drawing social reach from a probability distribution, but by assigning a probability to each possible connection. Hereby the probability decreases with increasing distance and increasing accumulated degree of the two nodes to be connected. Hence, we favor the attachment of new edges between nodes that have been unconnected or sparsely connected before. This may seem to oppose the assortativity assumption. However, isolated and sparsely connected nodes within the data set do not necessarily indicate that those individuals are disconnected in reality but rather that their friendship nominations have been outside their school class and are thus missing information. By controlling the probability of attaching a new link by the accumulated degree of two nodes, we favor the attachment of new links to nodes that feature not at random missing information (MNAR, defined in previous Section) about friendship ties. This operator differs from the “Waxman Model”, where the density of the links is controlled by a global parameter. Although this approach is very close to the “Waxman Model”, we address it with the term “social circles approach” in the remainder of this paper, because the use of the distance between pupils has been inspired by Hamill and Gilbert’s social circle model. For every possible connection that does not yet exist, we execute the following procedures: A connection probability is computed according to Eq 1. Hereby *P*_*link*_ denotes the connection probability; *d* stands for the distance in meters between the two nodes; *k* denotes the accumulated number of neighbors of both nodes and *c* is an adjustable parameter.

$$\begin{array}{c} P_{link}\left(u,v\right)=e^{\frac{-d(u,v)k}{c}} \end{array}$$ (1)

As *P*_*link*_ is being affected by the accumulated degree of the respective vertices, the order of link-estimation matters. In order to favor links between close vertices, *P*_*link*_(*u*, *v*) is being calculated for randomly picked *u* and a semi-random *v*. Semi-random in this case means that *v* is chosen randomly among the vertices of the closest school to *u* that has not been chosen yet. After computing *P*_*link*_, a random threshold *r* ∈ [0, 1] is generated for each possible link. If *P*_*link*_ surpasses the threshold, the new connection is effectively created.

#### Cold start link-prediction—Bootstrapping approach

The described social circle approach makes use of the “social distance” between two individuals and, implementing the circle concept, implicitly generates expected network properties. However, it does not make use of information that is implicitly stored in the network structure. We for example know that transitivity or triadic closure is a common phenomenon in social networks. This means that the probability that an edge exists between two nodes increases with the number of common neighbors of those nodes. In order to use this implicitly available information for creating the missing links between the isolated components of our network data, we implement as a second approach a two phase bootstrapping algorithm as proposed in [20]. The two phase approach allows for estimating probabilities for all eventually existing and not yet nominated friendships in the network and subsequently applying graph based measures to reinforce the probabilities for the existence of links.

**Phase I** makes use of individually available information about the nodes, according to the work of Leroy et. al. [20]. Yet, Leroy et. al. deal with data from Flickr. Thus, they use the common membership in thematic groups of two individuals to estimate a probability for the existence of a link between them in the first phase. In the underlying case, information is available about the district of the domicile and of the school of the pupils. We also posses information about leisure activities such as membership in sports associations or religious organizations of the children, about their integration into their neighborhood and how they get to school. We claim that friendships between children that emerge outside the school environment are frequently established either in the neighborhood of the domicile, during leisure activities or on the way to school. We therefore use this information to estimate probabilities for the existence of links that have not been recorded within the network survey conducted at class level. Similar to Leroy et. al, we define groups as co-occurrence of geographical and behavioral properties of the nodes, such as doing the same activity within the same district, or sharing a means of public transport on the way to school.

In order to assign probabilities *P*_*link*_1*γ*__(*u*, *v*|*u*, *v* ∈ *γ*) for the existence of a friendship between two individuals that share a group *γ*, we compute the share of links between the nodes that belong to the respective common group *γ* within the original data according to Eq 2. Hereby *e*_*γ*_ denotes the number of links originating from vertices within the group and *l*_*γ*_ denotes the number of links between vertices within the group. Please note that *e*_*γ*_ also contains links to vertices outside the group.

$$\begin{array}{c} P_{link_{1\gamma }}\left(u,v\right)=\left\{\begin{array}{cc} \frac{l_{\gamma }}{e_{\gamma }}, & if\;u,v\in \gamma .\\ 0, & otherwise. \end{array}\right. \end{array}$$ (2)

Considering that the membership in each common group may be the cause for the friendship between the two individuals in question, the outcome “friendship” occurs if a link is established within at least one of the common groups (*γ*) of the two individuals. Hence to estimate the existence of a friendship, we compute the probability that the outcome “friendship” occurs at least once within a set of outcomes *q* of size 2^*k*^ − 1, where *k* is the number of distinct common groups (*γ*). Each outcome has the from (*x*_1*q*_, …*x*_*kq*_), where *x*_*kq*_ ∈ [0, 1], here *x*_*kq*_ = 1 indicates that a link was established within the respective group (*γ*) and is assigned the probability *P*_*link*_1*γ*__(*u*, *v*). The probability for *x*_*kq*_ = 0 is accordingly 1 − *P*_*link*_1*γ*__(*u*, *v*). As an example, if two individuals shared exactly two groups, the set of outcomes that cause the establishment of a friendship between the individuals would consist of the outcomes (1, 0), (0, 1), (1, 1). The missing outcome (0, 0) would not lead to a friendship between them, as they here neither met in the first, nor in the second common group. The total probability for the existence of a link between a pair of nodes (*u*, *v*) can then be computed as the sum of the probabilities provided by each outcome within the set of outcomes *q* according to Eq 3. All groups used may be reviewed in Table 1. The respective probabilities *P*_*link*_1*γ*__(*u*, *v*|*u*, *v* ∈ *γ*) vary significantly with group size. Thus, *P*_*link*_1*γ*__(*u*, *v*|*u*, *v* ∈ *γ*) increases significantly with decreasing group size. A link between two individuals from a small district with few other pupils that share a common activity is hence more likely than a link between two individuals from a large district.

**Table 1:** Group definition.

Name	Condition
Sports	Pupils live in the same district and regularly practice sports
Church	Pupils live in the same district and frequent church or religious services
Transport	Pupils live in the same district, go to school in the same district and use the same public transportation

The table presents combinations of activities and locations that define a group. Pupils that share a group are assigned a certain probability that a link exists between them.

We recognize that drawing *P*_*link*_1*γ*__(*u*, *v*|*u*, *v* ∈ *γ*) from the observable data may introduce bias, as we can only observe friendships between pupils that visit the same class. We believe however that common groups and common interests play a major role in the establishment of friendships also on class level and hence consider the available data as a good indicator for the weight of common groups on link creation.

$$\begin{array}{c} P_{link_{1}}\left(u,v\right)=\sum_{i=1}^{2^{k}-1}\prod_{q=1}^{k}p\left(x_{iq}\right) \end{array}$$ (3)

**Phase II** foresees the application of graph-based measures. “*Common_Neighbors*” [10] measure has been shown to perform well as graph based measure for reinforcing probabilities of links that will eventually exist in the second phase of the bootstrapping algorithm [20]. We therefore implement the adaption of “*Common_Neighbors*” for the cold-start link-prediction problem according to [20]. Consequently we derive probability scores *score*(*u*, *v*) adding the probability *P*_*link*_2__(*u*, *v*) derived from “*Common_Neighbors*” measure to the probability *P*_*link*_1__(*u*, *v*) calculated in **Phase I** of the bootstrapping method. *P*_*link*_2__(*u*, *v*) is hereby computed according to Eq 4 for the pair of nodes (*u*, *v*) as the sum of the probabilities of each node *y* within the graph *U* being linked to both, *u* and *v*.

$$\begin{array}{c} P_{link_{2}}\left(u,v\right)=\sum_{y\in U}P_{link_{1}}\left(u,y\right)\times P_{link_{1}}\left(v,y\right) \end{array}$$ (4)

Subsequently, the scores calculated in **Phase I** and **Phase II** are converted to probability values inline with the work of Leroy et. al. [20] using a simple logarithmic function as presented in Eq 6.

$$\begin{array}{c} score\left(u,v\right)=P_{link_{1}}\left(u,v\right)+P_{link_{2}}\left(u,v\right) \end{array}$$ (5)

$$\begin{array}{c} P_{link}\left(u,v\right)=\frac{log(score(u,v)+1)}{log(max(score(u,v)|u,v\in U)+1)} \end{array}$$ (6)

In order to define the links that finally exist, we apply a threshold *r* ∈ [0, 1]. Links that have been estimated to exist with a probability *P*_*link*_(*u*, *v*) ≥ *r* are considered as existing links.

#### Combined approach

The bootstrapping approach employs more information about the nodes than the social circle approach does. Hence we expect that individual link-prediction is more accurate with the bootstrapping approach. However, the effectiveness of this approach is restricted by availability and granularity of information. In our implementation, for example, we define groups based on the district of residence of a pupil and his personal activities. This is a very strong restriction as it implies that friendships between pupils from adjacent districts are not considered. Hence the granularity of available information limits the effectiveness of the bootstrapping approach. To overcome these restrictions, we create a combined approach aiming at joining the generality of the social circles approach with the specificity of the bootstrapping method. For this purpose, we incorporate the social circles approach in the first phase of the bootstrapping algorithm.

In **Phase I** we slightly modify the calculation of *P*_*link*_1__(*u*, *v*) according to Eq 7. Other than in the social circles implementation, newly estimated links are not treated as existing links immediately, but receive a probability as in the Bootstrapping approach. Hence *k* may not be calculated as the degree of existing links of a node, but as the sum of probabilities of eventually existing links.

$$\begin{array}{c} P_{link}\left(u,v\right)=e^{\frac{-d(u,v)}{c}}*\frac{1}{k+1} \end{array}$$ (7)

Hereby we include personal information (group membership) into the definition of social distance according to Eq 8 where *x* and *y* describe the geographical vectors and *s* describes the social position of the individual. The social position is defined randomly on an interval $\left[0,\frac{\alpha }{2^{n}}\right]$, where *n* is the number of social activities of an individual. For experiments we used the social activities described in Table 1. We make sure that the social distance between two individuals declines with the number of common social activities.

$$\begin{array}{c} d\left(u,v\right)=\sqrt{{\left(x_{u}-x_{v}\right)}^{2}+{\left(y_{u}-y_{v}\right)}^{2}+{\left(s_{u}-s_{v}\right)}^{2}} \end{array}$$ (8)

**Phase II** implements the “*Common_Neighbors*” measure as explained in the bootstrapping approach. Subsequently we generate a random threshold *t* ∈ [0, 1] for each possible link. If *P*_*link*_(*u*, *v*) surpasses the threshold, the new connection is effectively created.

## Results

In this Section we explain how the quality of the generated global networks is measured and present the experimental results for the network extrapolation techniques *Social Circle Approach*, *Cold Start Link-Prediction* and *Combined Approach*.

### Quality assessment

In order to assess the quality of the implemented extrapolation techniques, we introduce a set of reference values in a first step. For assessing how well the generated global network represents properties of real world social networks, we compare the generated values for a number of network measures. The first measure is the *average degree* of the network, calculated as the average number of friends, the individuals within the network have. We further consult the network *density*, given by the ratio of links to the number of possible links within the network. *Average Shortest Path* indicates the average number of steps needed to reach any node *v* when starting from any node *u* in the network, if a path exists between them. We calculate the *Clustering Coefficient* as the ratio of the triangles and connected triplets in the graph. Moreover, *Assortativity* indicates the correlation between the degree of connected vertices as proposed in [31]. Finally, we consider the average number of links a student has outside his school environment *Out-links* as a reference value. We draw our reference values from distinct sources in the literature. The respective benchmarks and sources may be reviewed in Table 2.

**Table 2:** Reference values.

reference value	objective range or limit	Description
*Average Degree*	∈ [5, 10] (objective range)	We consider networks of close friendships, hence the average degree may be limited [22].
*Density*	≤ 0.014	This density value has been calculated for a larger but similar study with adolescents in the United States [40]. However, as we interconnect the isolated schools we expect a lower density value.
*Average Shortest* Path	∈ [5, 7] (objective range)	We expect “small-world” features [23].
*Clustering Coefficient*	≤ 0.252	This clustering value has been calculated for a larger but similar study with adolescents in the United States [40]. As we interconnect the isolated schools, where we expect to have less connections between schools than within schools, we expect a lower Clustering Coefficient.
*Assortativity*	*positive*	The positive assortativity indicates the existence of preferential attachment [23].
*# Out-links*	∈ [1, 2] (objective range)	Average number of links outside the school in AddHealth study [22].
*# Isolated vertices*	min	We aim to connect isolated vertices and hence desire a minimum number of disconnected vertices.
*# Components*	min	We aim to connect components and hence desire a minimum number of disconnected components.

The table contains the measures chosen to evaluate the proximity of the generated networks to real world social networks.

We further assess the performance of the distinct parameter settings analyzing the degree distributions of the generated networks in comparison with real friendship networks, as well as the relation between probability of friendship and physical distance between any two individuals.

#### Social circles approach

Experiments with the social circles approach were run using different values for the parameter *c*. As the parameter *c* reduces the exponent of the exponential decay function in Eq 1 and hereby increases the probability for a new link to be formed, we expect to generate more highly connected networks with increasing values for *c*. Due to the spacial emphasis of this approach, very distant schools led to problems in the implementation, that is why these experiments have been run on a reduced data set, where outlier schools have been removed.

**Fig 2:**
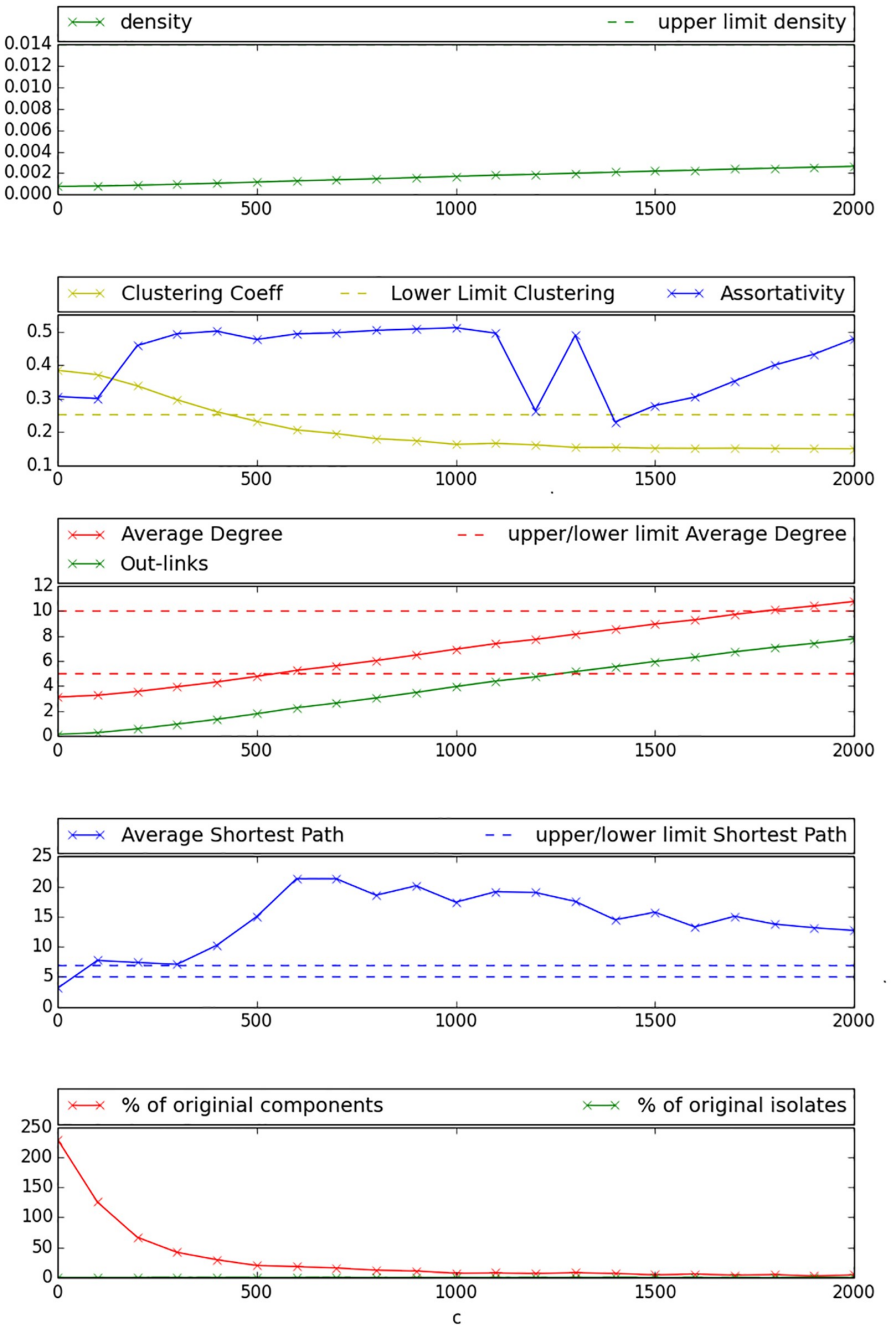
Social circles—Objective values. The abscissa scales the different values of the parameter *c* that controls the exponent of the exponential decay function in Eq 1; objective ranges and upper-/lower limits are indicated by dashed lines.

**Fig 3:**
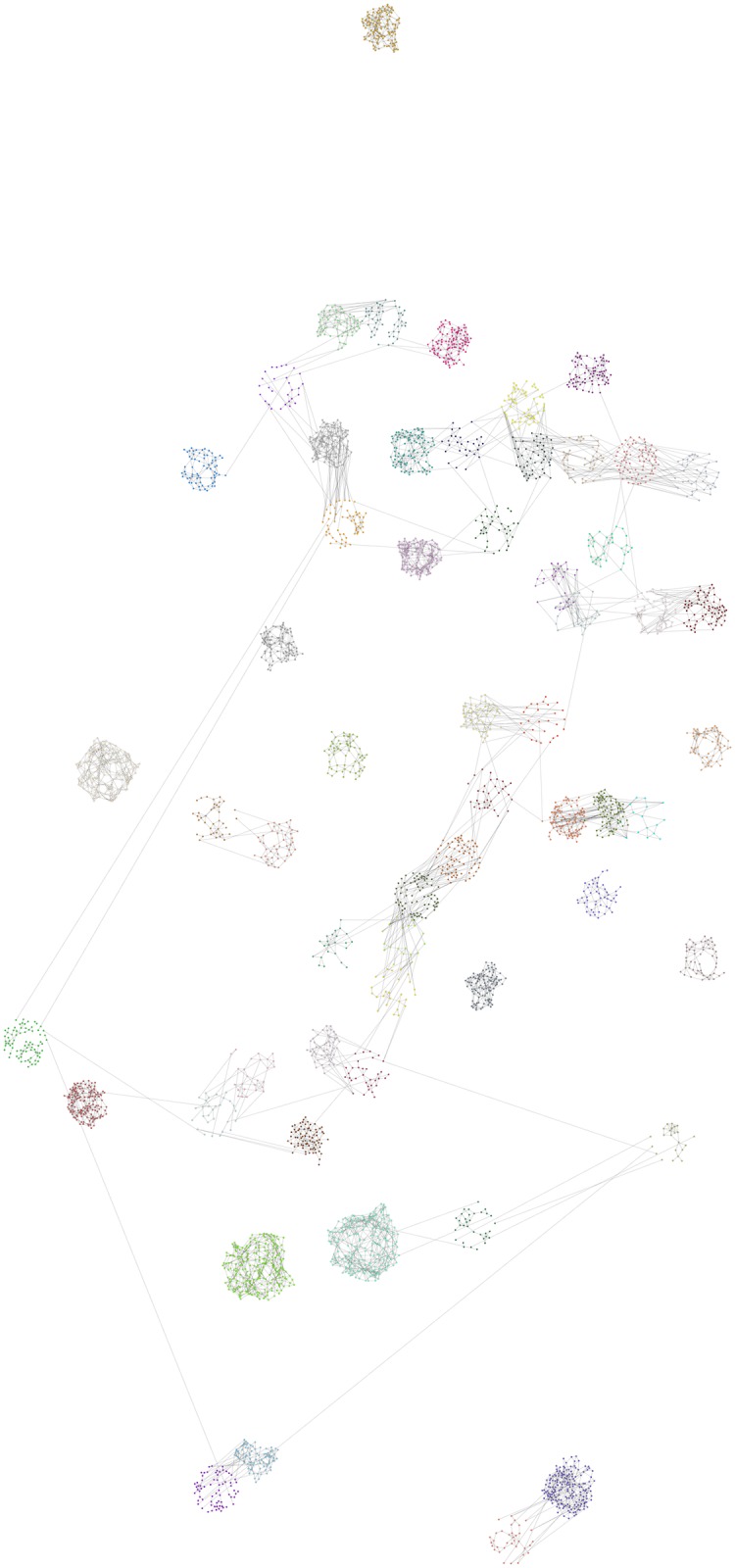
Network created by social circles approach. The Figure presents the individual pupils that participated in the FUNDAJ-Survey. Pupils are colored according to their school. Location of pupils is assigned by a Fruchtermann-Reingold algorithm within a radius of *n*^2^ around the location of their school within the city of Recife. *n* indicates the number of pupils of the respective school. Grey lines indicate friendships between pupils as registered by the survey, as well as friendships estimated by the social circles method applying *c* = 500.

Experimental results for the reference values pointed out in Table 2 are illustrated in Fig 2. One may observe that *density* indicated by the green solid line in the upper Figure takes values between 0.001 and 0.003 for all networks generated with variations of parameter *c*. It hereby lies steadily under the maximum value indicated in Table 2. The blue solid line in the second sub-figure representing the networks assortativity-coefficient fluctuates for different *c* values but remains positive for all parameter settings. The *Clustering-Coefficient* as represented by the yellow solid line in the second sub-figure decreases with increasing *c* and crosses the upper limit defined in Table 2 at a *c* value of approximately 500. The number of *Out-links* increases with growing *c* as presented by the solid green line in the third sub-figure, ranging from 0 for low *c* values to 8 for very high *c* values. *Average Degree* is being illustrated by the solid red line in the third sub-figure. It increases also with increasing *c* and reaches the objective range as indicated in Table 2 between the two dashed red lines for *c* values between 500 and 1800. *Average Shortest Path*, represented by the blue solid line in the fourth sub-figure, raises for low *c* but subsequently decreases with increasing *c*. This seems logical, since shortest path is calculated as the average of the shortest paths of all components in the graph. As the components become better interconnected with growing c, shortest path initially increases. Predefined objective interval for *Average Shortest Path* as indicated by the blue dashed lines can be reached for *c* values below 400. The last sub-figure illustrates the results for the number of components as a percentage of original number of components with a red solid line, and the total number of isolated vertices as a percentage of original number of isolates with a green solid line. It can be observed that the percentage of isolated nodes can be kept close to zero for all *c* values, while the percentage of components decreases with increasing *c* and reaches values close to zero when applying *c* values of 500 or higher. The Figure presents percentages above 100% for very few values of *c*. This is possible as components are defined as a set of at least two connected nodes that are only linked to each other. Hence, for those very low *c* values many former isolated nodes connect to each other and form new components. Fig 3 presents the generated network with a *c* value of 500, where colored points represent pupils (each school is indicated by a different color) and edges represent friendships between the pupils. This Figure illustrates that a globally interconnected network has been generated where social network typical patterns can be observed.

#### Cold start link-prediction—Bootstrapping approach

The bootstrapping approach has been implemented according to the description in the previous Section. The respective experiments were carried out with several values for the threshold *r* ∈ [0, 1]. The threshold *r* controls the probability that a link exists, thus we aimed to create more densely connected networks with decreasing *r*.

**Fig 4:**
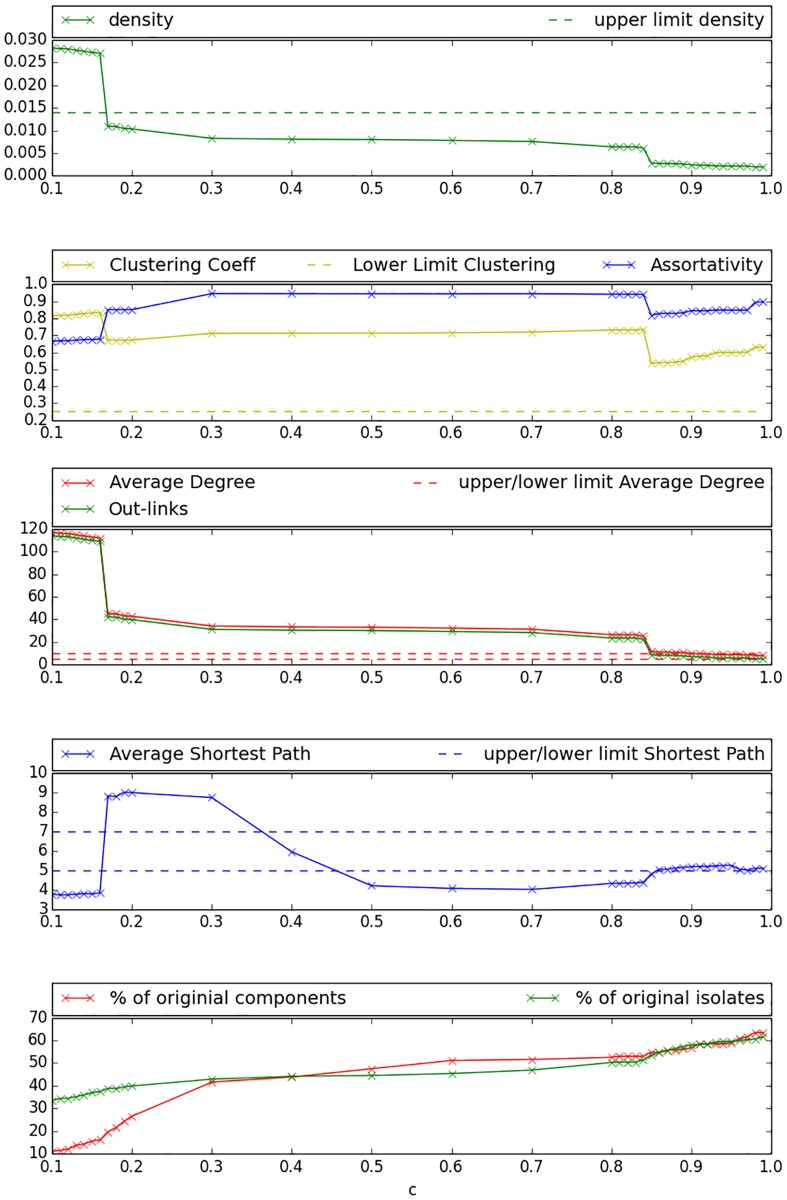
Bootstrapping objective values. The abscissa scales the different values of the threshold parameter *r*; objective ranges and upper-/lower limits are indicated by dashed lines.

**Fig 5:**
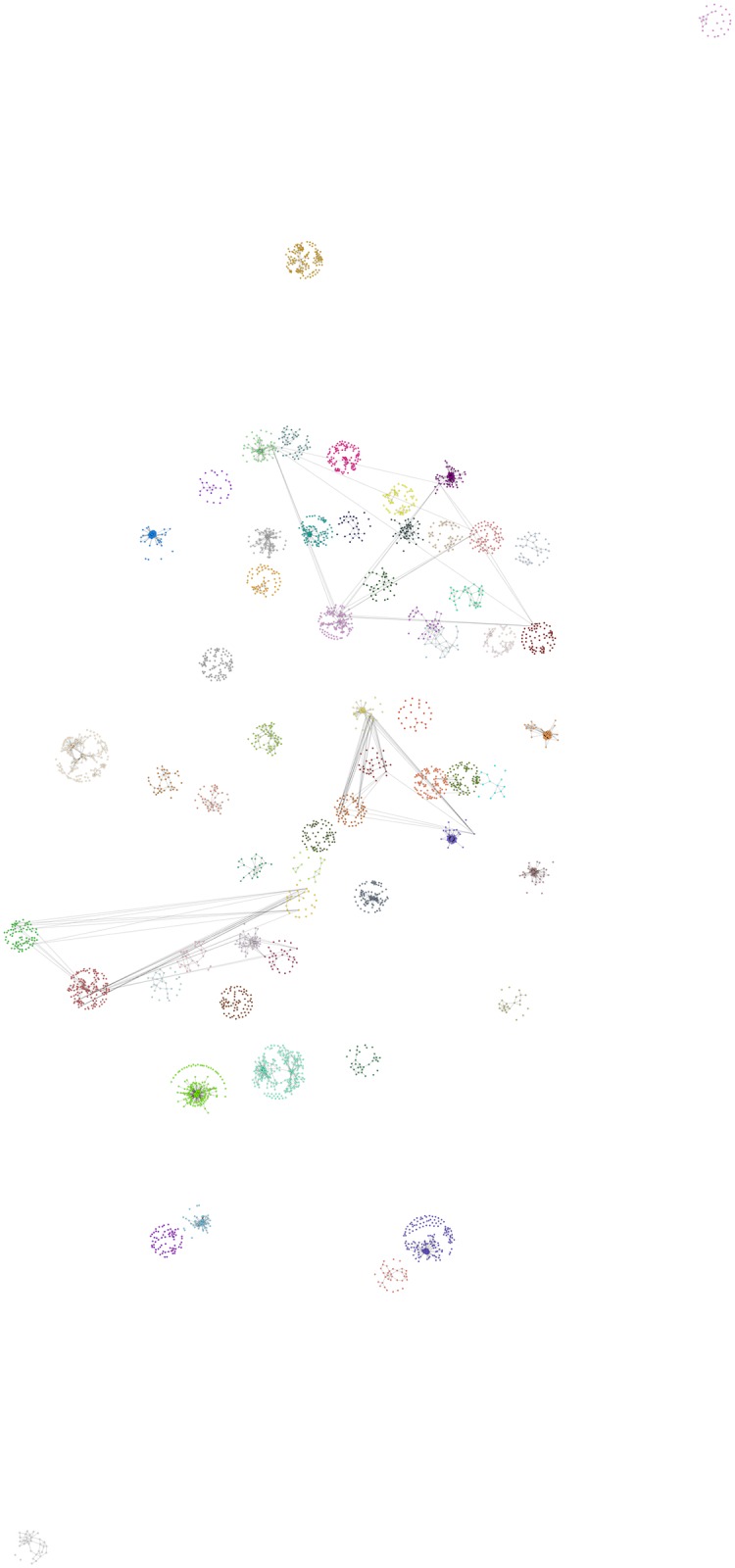
Network created by bootstrapping. The Figure presents the individual pupils that participated in the FUNDAJ-Survey. Pupils are colored according to their school. Location of pupils is assigned by a Fruchtermann-Reingold algorithm within a radius of *n*^2^ around the location of their school within the city of Recife. *n* indicates the number of pupils of the respective school. Grey lines indicate friendships between pupils as registered by the survey, as well as friendships estimated by the Bootstrapping method applying *r* = 0.91.

Fig 4 reveals in the first and second sub-figure that *Assortativity* indicated by the blue solid line remains positive for all settings, *Clustering Coefficient* as indicated by the yellow solid line remains far above the maximum value and *density* represented by the green solid line remains below the maximum value for most settings. However, the third sub-figure shows that *Average Degree* and *Out-links* reach undesirably high values for low settings of *r* and can only reach the objective area for experiments with *r* ≥ 0.89. As indicated by sub-figure four, *Average Shortest Path* reaches desired values for *r* ≥ 0.89. Analysis of sub-figure five yields that low and therefore desirable percentage values for the number of isolated components and the number of isolated nodes can be reached for low *r*. However, those indicators still yield a reduction of more than 30% for settings with high *r*. Fig 5 illustrates the network created with the bootstrapping technique using a threshold of 0.91. One may observe within this Figure that the created network still contains a considerable amount of isolated components and that many schools remain unconnected even if they are very close to other schools. Furthermore, connections seem to be established between very few individuals of the different schools.

#### Combined approach

As explained in the previous Section, the combined approach incorporates elements from the Social Circles and Bootstrapping approaches. Experiments were run using varying values for the parameter *c* that controls the probability for a new connection to exist in the first phase of the algorithm. Since a random threshold is assigned to each connection finally, results can be illustrated by scaling the values for parameter *c* on the abscissa.

**Fig 6:**
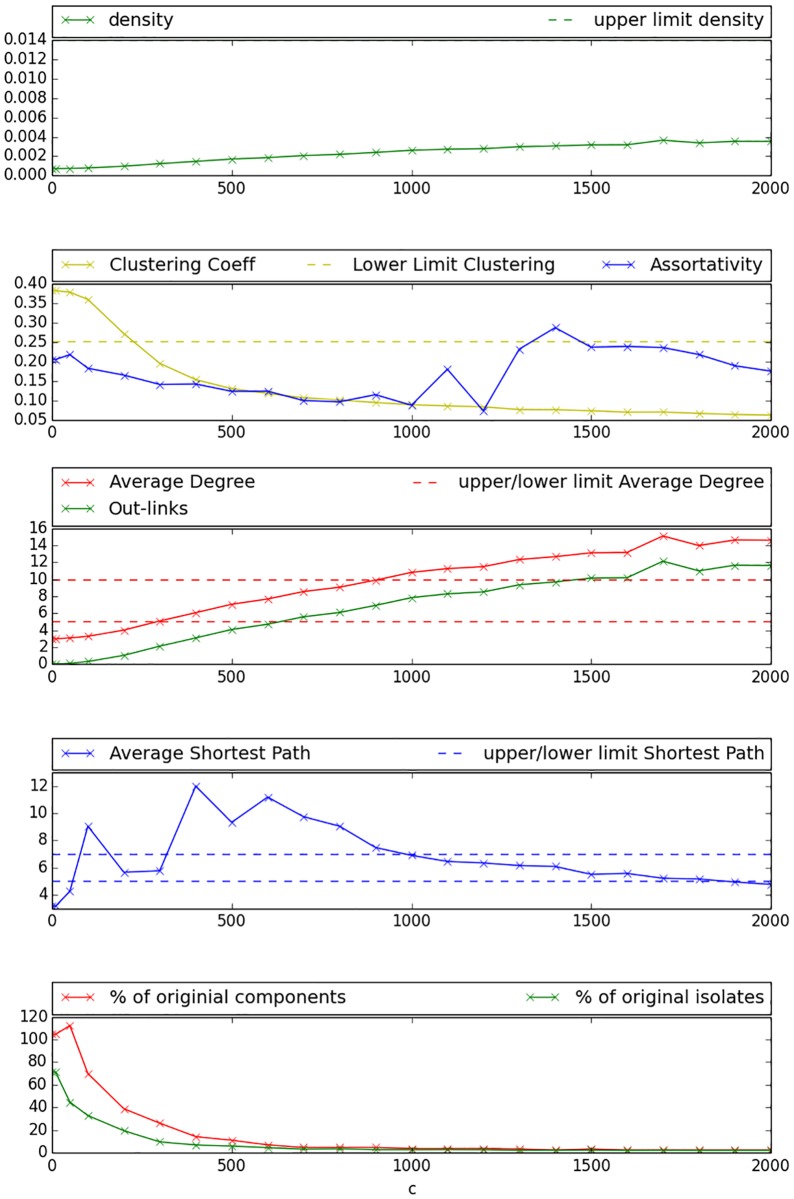
Combined approach objective values. The abscissa scales the different values of the parameter *c* that controls the exponent of the exponential decay function in Eq 7; objective ranges and upper-/lower limits are indicated by dashed lines.

**Fig 7:**
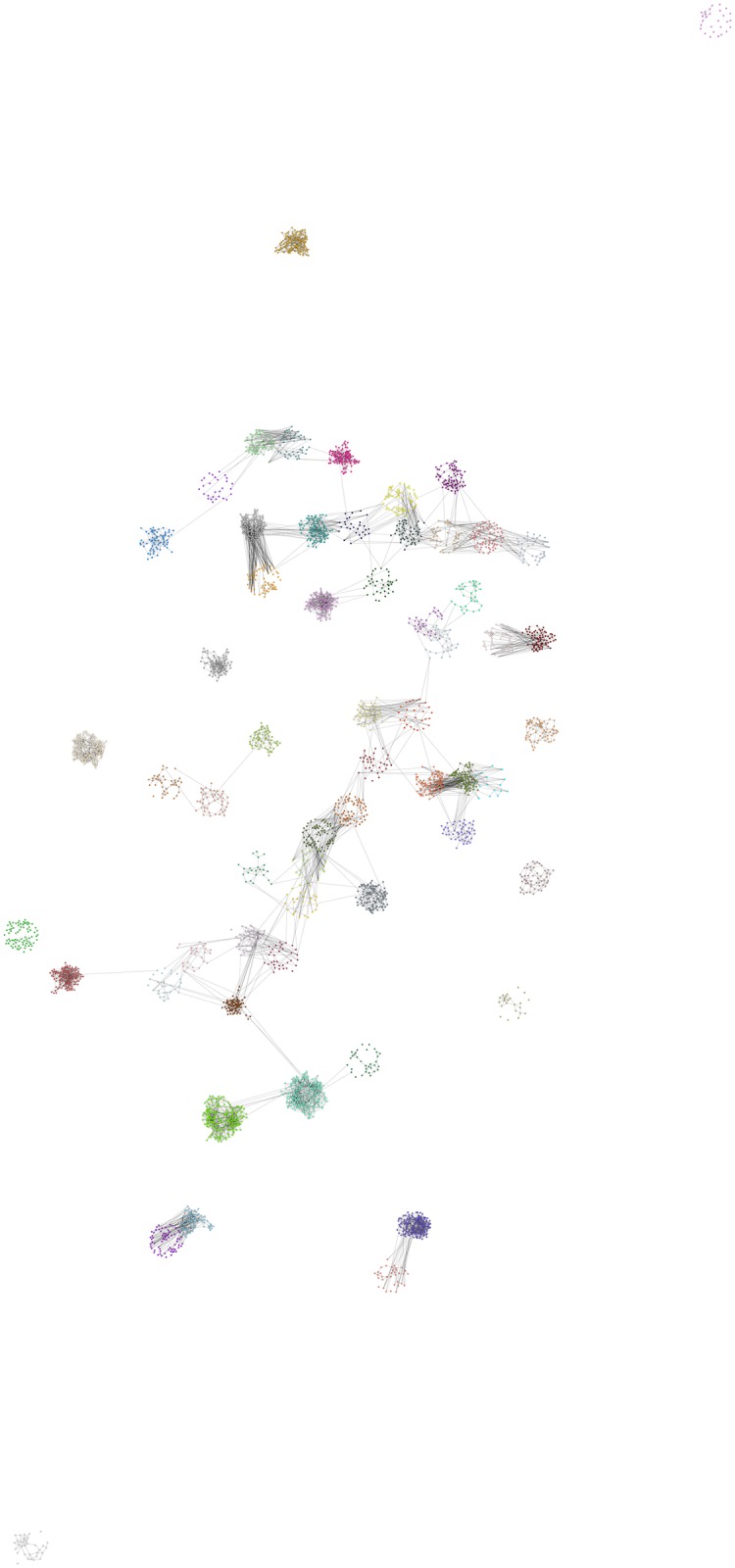
Network created by combined approach. The Figure presents the individual pupils that participated in the FUNDAJ-Survey. Pupils are colored according to their school. Location of pupils is assigned by a Fruchtermann-Reingold algorithm within a radius of *n*^2^ around the location of their school within the city of Recife. *n* indicates the number of pupils of the respective school. Grey lines indicate friendships between pupils as registered by the survey, as well as friendships estimated by the combined method applying *c* = 300.

As Fig 6 shows in the first sub-figure, density can be kept on a desirable level for all *c* values. The second sub-figure reveals that also *Assortativity* remains positive for all *c* values, but *Clustering Coefficient* declines for growing *c* and reaches values under the objective upper limit for *c* ≥ 200. We observe in the third sub-figure that values for *Average Degree* reach the objective range for *c* ∈ [300, 900], while this can be stated for *Out-links* for *c* ∈ [0, 300]. *Average Shortest Path* as shown in the fourth sub-figure remains within the objective range for nearly all values of *c*, except *c* ∈ [300, 900], where the objective range is slightly exceeded. As illustrated by the fifth sub-figure, the number of isolated components and isolated nodes decreases steeply with growing *c*, reaching an almost totally interconnected network for *c* ≥ 600. Fig 7 presents a network generated with the combined approach under a *c* value of 300. Obviously, inter-school links are better distributed between pupils and the graph seems better inter connected than the comparable graph generated with the bootstrapping approach. However, the network remains not fully connected for this setting and especially more distant schools remain isolated.

#### Analysis of degree distribution

The measures introduced in the precedent Sections indicate that all three approaches where capable of generating networks that exhibit real world social network properties. In order to better understand the outcomes for the different approaches, this subsection analyzes the degree distribution of the generated networks in relation to degree distributions of real world social networks. For comparison we plot the original degree distribution of the FUNDAJ graph, the degree distribution of the AddHealth Network and the in- and out- degree distributions of the testimonial network of the Korean online social network “Cyworld”. The National Longitudinal Study of Adolescent to Adult Health (AddHealth) network was surveyed among a representative sample of adolescents in grades 7 to 12, it contains 90118 students from 145 schools in 80 communities in the United States of America. Students could not only nominate peers from their own school, but also peers from a “sister-school”. Add Health combines longitudinal survey data on respondents’ social, economic, psychological and physical well-being with contextual data on the family, neighborhood, community, school, friendships, peer groups, and romantic relationships [40].

**Fig 8:**
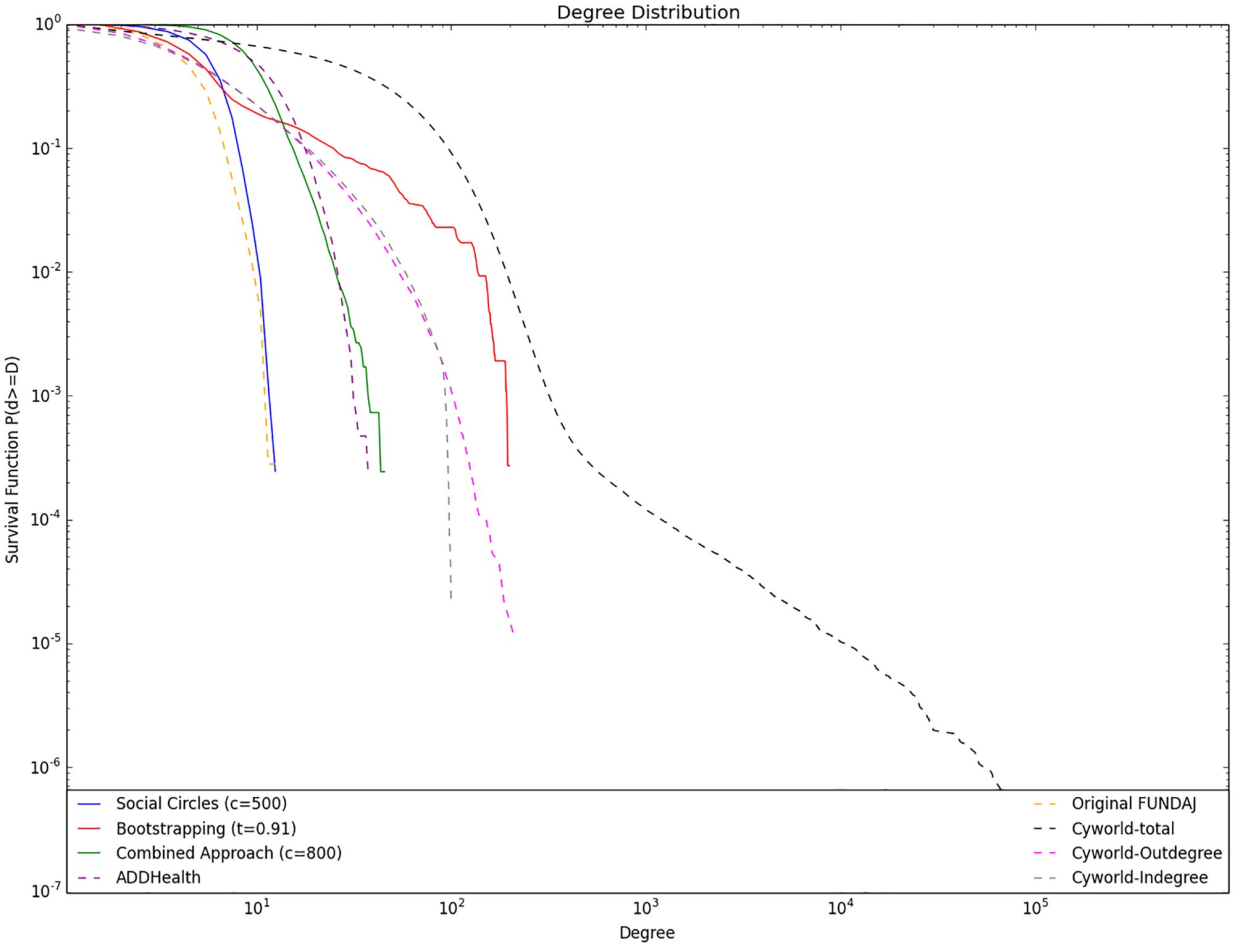
Degree distributions for networks generated with social circles (c = 500), bootstrapping(r = 0.91) and combined approach (c = 800). Compared to degree distributions from real world social networks: Original FUNDAJ Graph, AddHealth Network and Cyworld in-and out degree distribution. Social Circles Approach maintains the original Degree Distribution, Combined Approach approximates the AddHealth Degree Distribution and Bootstrapping approach generates a network similar to Cyworld online social network.

Cyworld is an online social network similar to Facebook, where users may create a personal profile and create friendship ties to other users. Users are hereby enabled to write testimonials for other users. As the writing of a testimonial requires some effort and knowledge about the receiver of the testimonial, it has been shown that the network of testimonials in Cyworld resembles real friendship networks very closely [41]. In the remainder of this paper, we examine exclusively the Cyworld testimonial network, however in order to ensure readability, we refer to it as the “Cyworld network”. Fig 8 illustrates the degree distributions of the networks generated with the presented techniques along with log-log plots of degree distributions of Cyworld-, Add-Health- and the original FUNDAJ Network. Here the solid lines represent networks that were extrapolated based on the FUNDAJ graph. The dashed lines indicate the real networks. The Figure reveals that the different approaches generate degree distributions similar to the distributions observed in real friendship networks. It is striking that the degree distribution generated by the social circles approach, as indicated by the blue solid line, appears to very closely approximate the original FUNDAJ graph, represented by the dashed purple line, featuring a similar shape and comparable maximum degree. On the other hand, the AddHealth-degree distribution (dashed turquoise line) seems to be well represented by the combined approach (solid red line) as their plots are almost congruent. Finally, the complementary cumulative distribution (CCDF) of Cyworld in- and out- degrees can be well approximated by the bootstrapping approach, as shown by the obvious similarity of the graph indicated by the green solid line (Bootstrapping approach) and the dashed yellow and black lines that represent Cyworld out- and respectively in- degree distributions.

#### Analysis of friendship probability in relation to physical distance

**Fig 9:**
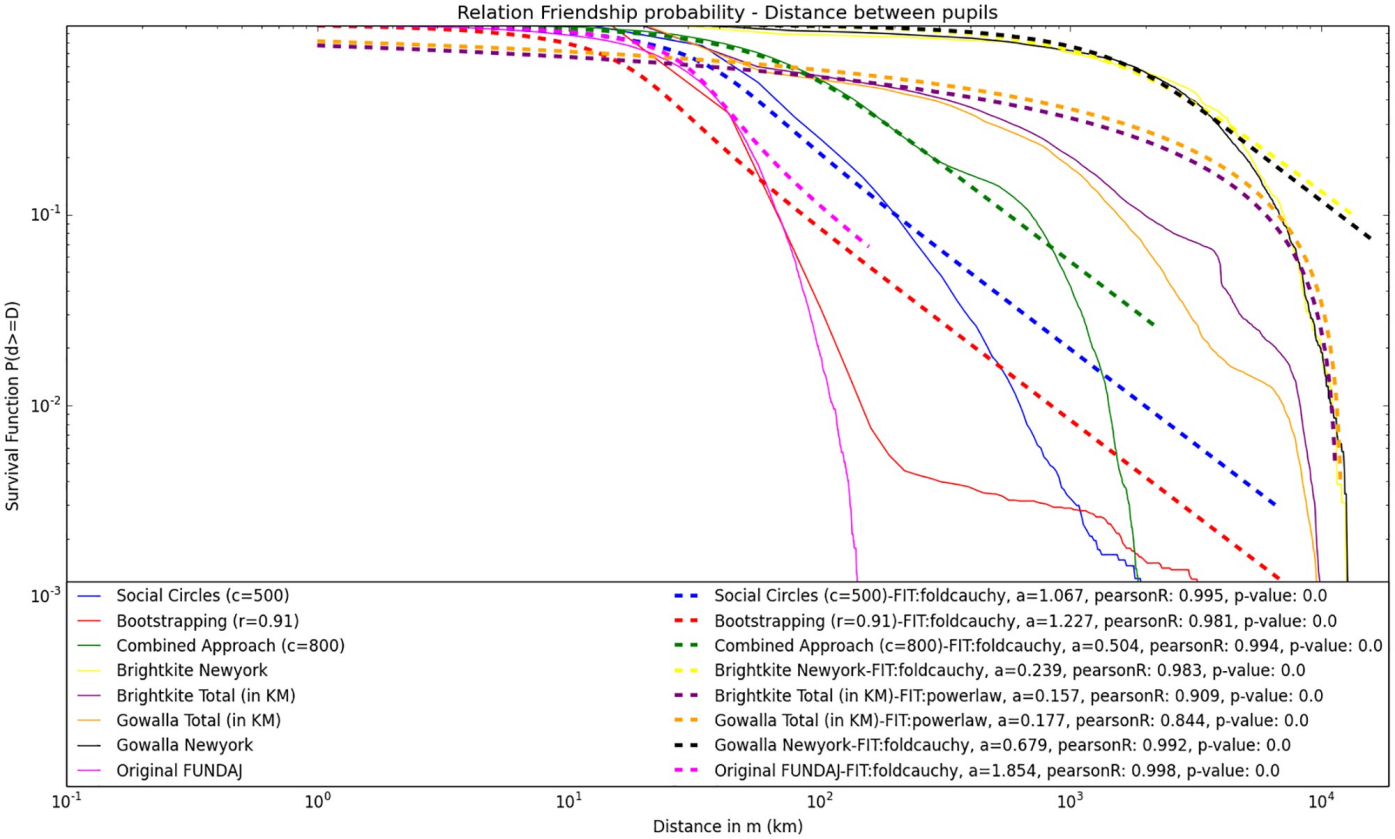
Log-Log plot of survival function (CCDF). Link-probability related to physical distance between nodes for networks generated with Social Circles (c = 500), Bootstrapping(r = 0.91) and Combined approach (c = 800). Compared to distance- link-probability distributions from real world social networks: Brightkite and Gowalla worldwide networks, as well as local sub networks for the city of New York.

Another characteristic property of real world friendship networks is the relation between link-probability and distance between nodes [26]. It seems intuitive and is also universally agreed that the probability for the existence of a link decreases with increasing distance between the respective nodes. This property can also be found in recent networks, despite the progress made in information and transportation technology, while the exact relation varies depending on the studied data set [26]. Therefore, if our generated networks are to represent real-world features, we expect to find a similar relationship for distance and node probability within the extrapolated data. Hence, in order to evaluate the suitability of our approaches to produce real-world network properties, Fig 9 contrasts CCDF log-log plots for the probability of the existence of a link between nodes within a certain distance for the networks generated with the presented techniques, as well as from real world social networks. Here the solid lines represent either distributions from networks that were generated using the presented techniques, or distributions from the location-based online social networks Brightkite and Gowalla (purple and orange solid lines). Both, Brightkite and Gowalla are location based online networking services, that enable the user not only to establish links to other users, but also to provide information about his current location. These characteristics make of Brightkite and Gowalla good references for the analysis of friendship probability in relation to physical distance in extrapolated social network data. Data from Gowalla and Brightkite where obtained from the Stanford Large Network Dataset Collection [39]. As both data sources provide time series information about the places, the users stayed at, we considered the most frequent location for each user as his respective domicile.

As the experiments presented in this paper all extrapolate the network data obtained by FUNDAJ in the city of Recife, comparison data was required from a locally restricted urban environment. Hence, we also plotted subsets of the Gowalla and Brightkite networks, containing solely individuals located within the city of New York (locations within the latitude interval [40.65, 40.80)] and the longitude interval [−74.05, −73.90]). The solid yellow line represents the distribution for the Brightkite network from New York, while the black solid line indicates the Gowalla New York network (yellow and black lines are very close). In order to compare the different distributions, a total of 90 continuous probability distributions from the Numpy Library [42] were fitted to the data via least squares fitting. The distribution with the highest p-value and lowest Kolmogorov-Smirnov test statistic was chosen as the best fit.

Fig 9 reveals that all local networks could be reasonably well represented by folded Cauchy distributions (as presented in Eq 9) with slightly different values for parameter *a*, while the distributions of the global Brightkite and Gowalla networks seem to be better approximated by a power law distribution with exponent between 0.15 and 0.7.

$$\begin{array}{c} f\left(x,c\right)=\frac{1}{\pi *(1+{\left(x-a\right)}^{2})}+\frac{1}{\pi *(1+{\left(x+a\right)}^{2})} \end{array}$$ (9)

Please note that the survival function of the power law plot does not appear as a straight line in this graph, as it is usually the case for power law relations on log-log pots. This is due to the finite nature of distances on earth. As distances between individuals on this planet are restricted, the power law plot bends down as the *x*_*max*_ is approached.

Goodness of fit is also checked, calculating the Pearson Correlation Coefficient for each pair of distribution and fit. The correlation coefficient supports the goodness of fit for all distributions and respective fitted distributions. Visual analysis reveals that the network generated using the combined approach approximated the Gowalla and Brightkite networks of the city of New York best, while Social Circles approach seems to better maintain the original distance-link-probability as indicated by the pink solid line (original graph) and the purple dashed line (fitted folded Cauchy distribution). The red solid curve that indicates the relation of distance to link-probability for the Bootstrapping Approach appears to be differently shaped than the other distributions, as it shows a buckle for distances greater than 10^2.5^. The correlation coefficient still suggests the folded Cauchy distribution with parameter *a* = 0.659 as a good fit for the Bootstrapping curve. This disturbance stems from the nature of log-log plotting, where the deviations scale down for large values of the variables. Further observation of parameters *c* of the fitted folded Cauchy distributions supports the hypothesis that the combined approach most closely approximates the local Gowalla and Brightkite data, as the fitted folded Cauchy distribution for the combined approach has parameter *a* = 0.504 and hence lies between Gowalla New York (*a* = 0.679) and Brightkite New York (*a* = 0.234). The fitted folded Cauchy distribution for the Bootstrapping Approach network exhibits a parameter (*a* = 0.659) even closer to Gowalla New York. However, due to the different shape of the curves this similarity should be taken carefully. Moreover, the parameters of the fitted folded Cauchy distributions for the original network (*a* = 1.854) and the Social Circles Approach (*a* = 1.067) seem to be quite similar.

## Discussion

This Section evaluates the results obtained from the experiments and sets them in the context of the initial problem, to extrapolate given data of social networks containing isolated components. We discuss the performance of the three presented approaches and point out promising applications for each of them. Subsequently, Table 3 summarizes the findings and recommendations for the application of the proposed techniques. The original data set solely contains the information of the position of the pupils on school level. It is hence known, where they go to school but not exactly where they live. This problem occurred because the survey participants could nominate any district of living. However, colloquial naming of districts differs significantly from the official denominations and a large number of participants gave rather descriptions of the locations than real addresses. Moreover, no information about links between students from different schools is available. As students are assigned a random location around the location of their school, the existing networks on school level currently cannot serve as test set for the location-based algorithms. Hence, assessment of precision is not possible within the underlying data set. Nevertheless, an evaluation of accuracy of predicted links would be desirable but as mentioned before, the aim of this study is rather the estimation of a good model of the global network but a very precise estimation. Thus, no assessment of precision has been carried out.

**Table 3:** Recommendations for application.

setting	Social Circles	Bootstrapping	Combined
*c* ≤ 500	Social network extrapolation for city networks, where acquaintances are locally restricted. Distant districts remain isolated. Due to missing transport, social divide or decentralized city architecture	-	-
500 ≤ *c* ≤ 1000	Extrapolation of social networks, where a globally connected network is to be created, sparse linking between dense local networks. “fixed choice effect” does not disturb the data e.g: close friendship network	-	Social network extrapolation within cities (*c* = 800 seems optimal). “fixed choice effect” and “boundary specification” are assumed to bias the original data.
*r* ≥ 0.89	-	may resemble online testimonial network, yet to be investigated	-
*r* ≤ 0.89	-	very high clustering. No conclusive social network.	

The table contains recommendations for the application of techniques and settings for distinct purposes.

### Social circles approach

The results presented in the previous Section indicate that most of the desired objective values presented in Table 2 can be reached with differing values of *c*. As the aim of this work is the interconnection of isolated sub-components of a given network, the number of components is a crucial value for demonstrating the suitability of our approach. Results indicate that a desirably small number of components may be reached using *c* values higher than or equal to 500. However, the trade-off between two benchmarks with opposing trends has to be analyzed more deeply: *Clustering-Coefficient* and *Average Shortest Path*. As shown in Fig 2, *Clustering Coefficient* reaches desirable values for *c* ≥ 500, yet *Average Shortest Path* reaches the objective zone for *c* values ≤ 300 and also *Out-links* exceeds the maximum value for *c* ≥ 500. Hence, one has to decide if it is more appropriate to create a network with “small world” properties but relatively high clustering and containing still a considerable number of isolated components or if a lower clustering, an interconnected network and relatively large values for *Average Shortest Path*, are desirable. In this case the researcher also assumes that the average number of links, an individual has with people from other entities is much higher than surveyed in the Add Health study [22].

The analysis of the degree distributions in Fig 8 shows that the Social Circles Approach maintains the original degree distribution. This indicates it as a good model to generate an interconnected network that takes only very close friends in consideration, while it incorporates the hypothesis that micro-structures repeat themselves also on the macro level.

Fig 9 illustrates that the Social Circles Approach generates the network exhibiting the highest similarity in distance-link-probability relation with the original graph. Even though maximum distance increases for the generated graph, the overall shape is kept and the overall structure of link-distances is maintained. We therefore argue that the Social Circles Approach extrapolates the network data, maintaining the original characteristics of the isolated components best.

### Bootstrapping approach

The results show that most objective values (social network properties defined in Table 2) can be met if running the cold start link-prediction approach using a high final threshold (*r* ≥ 0.89). This setting also led to an improvement in the interconnection of the network by reducing the number of isolated nodes and components by approximately 30%. However, it is not possible to create globally connected networks while reaching coherent values for average degree and the number of out-links of the pupils, because for those settings a considerable number of disconnected components remain. Moreover, clustering remains significantly above the lower limit defined in Table 2. This may stem from the restriction of the prediction of friendships to pupils that live within the same district. This is a very strict restriction that does not necessarily represent reality, as pupils that live close to the district border may also have contacts from adjacent districts. The threshold *r* needs to be set quite high in order to meet the objective values, impeding the creation of a globally connected network. This may be healed in a different scenario, where more information about the individuals is available and hence a larger number of common groups could be defined. The plot of the degree distribution of the network created by the Bootstrapping technique, applying a final threshold of (*r* ≥ 0.89), seems to approximate the in- and out- degree distribution of the Cyworld testimonial network. As mentioned in previous work [41], the Cyworld testimonial network has a few, very active testimonial writers with far above 100 written testimonials. The results produced here indicate that the restriction of activities to the four groups presented in Table 1 leads to a relatively small number of possibly connected individuals. Those individuals are assigned very high probabilities for link-creation in turn, and hence act similar to the “hubs” that appear in the Cyworld data. From that point of view the bootstrapping approach seems to be well suited to generate networks with a usual degree distribution for close friendship networks, incorporating the special feature of the described “hubs”. However, in terms of distance—link-probability relation, the generated network is not comparable to other location-based social networks, as the ill-shaped curve for the Bootstrapping graph in Fig 9 indicates. Nevertheless, the authors of this work do not have access to the location of Cyworld users, so it remains unclear if this ill-shaped curve could be a feature of online testimonial networks. On the other hand, the applied groups are very broad. this may lead to a quite homogeneous and discrete distribution of probabilities in the first phase. Hence, in order to improve the performance, one could also try to narrow the definition of groups and thereby decrease overall probabilities for the existence of common groups. This may also lead to a better performance of low thresholds.

### Combined approach

The combined approach yields a better reduction of isolated components than the cold-start link-prediction approach, while all other global measures can be kept within or close to the objective intervals. Those positive results can be obtained for parameter settings with 500 ≤ *c* ≤ 1000. The combined approach further approximates the degree distribution of the Add-Health school network very closely for *c* = 800 as shown in Fig 8. Although even the Add-Health study did not survey a completely interconnected network, but solely intra-school networks, it contains an interesting feature: the study design foresees a sister school for each participating school. The sister school is usually that junior-high school where the major share of students from a particular high school have studied before. The study designed allowed the surveyed pupils to nominate also friends from this sister school, which leads to a network that is better interconnected than the network surveyed by FUNDAJ. Moreover, the Add-Health questionnaire allowed the participants to nominate up to five best female friends and up to five best male friends, leading to a maximum of 10 nominated friends per pupil. Compared to the FUNDAJ survey, where only individual schools without sister schools have been surveyed and where a maximum number of five friends (female and male) could be nominated, this gives us a hint on how the “fixed choice effect” and “boundary specification problem” may influence degree distributions. The combined approach appears to be a good technique to extrapolate data in order to overcome those restrictions. Additionally, as presented in Fig 9, the combined approach generates a link-probability versus distance curve that approximates those distributions from location-based online social networks such as Gowalla and Brightkite, best. The approach shows therefore the most unambiguous results, indicating clearly which would be the best setting for this algorithm. However, the granularity of available information is still quite low. Recall that the social position of an individual is only defined by three activities, which is sports, church and using the same public transport method. The use of more detailed information could probably further improve the results.

## Conclusion

Within this work, we have investigated the suitability of three graph generation or respectively link-prediction techniques in order to impute [13] not at random missing data (MNAR) about social ties within social network studies, and to interconnect disconnected components within the surveyed network. Hereby we aimed at creating a model of the global network, stemming from original network data. The precision of the applied techniques was of secondary importance. Although a precise estimation of links would be desirable, we can settle with the creation of a reasonable model, especially because precision cannot be assessed with the available data set. Firstly we modified the Social Circles approach as proposed by Gilbert such that it was suited to impute missing links between the locally isolated components of the original graph (**Approach 1**). Secondly, we applied a bootstrapping approach proposed by Leroy et. al [20] (**Approach 2**) and thirdly we created a hybrid algorithm combining features from 1 and 2 (**Approach 3**). Our experiments show that all three approaches are able to impute data such that the number of isolated components within the graph decreases significantly, while the created networks exhibit properties that can be found in real world social networks.

We found that the three approaches create networks exhibiting different degree distributions that resemble degree distributions of different real world networks. While **Approach 1** appears to maintain the degree distributions of the original data set, **Approach 3** leads to a degree distribution that can also be found in more interconnected networks in the real world. Further, **Approach 2** was able to reproduce degree distributions of close online friendship networks. However, **Approach 2** was not able to create a completely interconnected network, when holding on to other restrictions such as a plausible average degree or clustering coefficient. Moreover, this approach led to an ill-shaped curve for the relation between link-probability and distance between two nodes. We could not find evidence for the existence of a curve with such a shape in real world networks. In this regard, **Approach 1** and **3** performed better, as **Approach 1** produced link-probability distance curves very similar to the original data set, while **Approach 3** was able to reproduce this relation very closely for the two locally restricted location-based online social networks Brightkite and Gowalla. We hence argue that the Social Circles **Approach 1** should be applied if the social scientist is willing to generate interconnected networks from unconnected components and acts upon the assumption that the macro network shall closely resemble micro structures. In other words, the approach put forward is able to predict links comprehensively if the assumption holds that missing data stems from “boundary specification” but that the “fixed choice effect” does not affect the data collection. An example for this could be the examination of best friends links between school children, where one assumes that most links are already established within the classroom and very few out links are missing. However, if one aims at creating interconnected networks, and does not expect that the network structure that can be observed in the original data approximates the macro network structure, the combined approach seems to be even more adequate. In this case not only “boundary specification” but also the “fixed choice effect” causes missing data. Results indicate that the bootstrapping approach enables the scientist to reproduce close online relations. However, more than the other approaches, this one requires additional information about the individuals and hence suffers from data granularity issues. Also, the combined approach may solely be suited for data sets that yield some individual information about the individuals, allowing for calculating a social distance between them. Moreover, it became clear that data granularity is a primary issue for the performance of the algorithms. The very general social circles approach seems to be very well suited for the task of completing a network of isolated components in a comprehensible way even if very little data about the nodes is available, or if this data is hard to structure for the underlying purpose.

Considering that even the large social network study that provided the data for this research only represents a relatively small sample of the whole population, it seems reasonable to settle for the good performance in generating networks that feature real world network characteristics. However, in combination with more information about the population (i.e, census data) the techniques that employ more personal information may contribute to an even more realistic network estimation.

Future work should deal with the application of the presented techniques to combine different data sources. In the underlying case, the survey data from FUNDAJ could hereby be connected to census data. In addition, further development of the social distance between a pair of nodes might improve performance of the combined approach and the social circles approach. Applied to the data set used within this research, this may lead to an even more plausible globally connected network. Further investigation of link-probability—distance curve of the bootstrapping network seems to be interesting, especially when employing more individual information about the nodes. In this case further comparison with data from online testimonial networks like Cyworld is recommended. The performance of the bootstrapping approach may be tested using more social groups with a more narrow definition. The additional groups may increase the quantity of generated links even for settings with a high threshold *r* and thereby heal the persisting problem of high clustering.

Additionally, testing the approaches on alternative data sets may give further insights regarding the precision of the applied techniques. Although for our purpose, precision is not essential, other applications may require proof of precision.
